# Psilocybin and the glutamatergic pathway: implications for the treatment of neuropsychiatric diseases

**DOI:** 10.1007/s43440-024-00660-y

**Published:** 2024-10-16

**Authors:** Izabela Szpręgiel, Agnieszka Bysiek

**Affiliations:** Department of Pharmacology and Brain Biostructure, Unit II, Maj Institute of Pharmacology, Polish Academy of Sciences, Smętna 12, Kraków, 31-343 Poland

**Keywords:** Psilocybin, Glutamate, GABA, 5-HT2A receptor, Depression

## Abstract

In recent decades, psilocybin has gained attention as a potential drug for several mental disorders. Clinical and preclinical studies have provided evidence that psilocybin can be used as a fast-acting antidepressant. However, the exact mechanisms of action of psilocybin have not been clearly defined. Data show that psilocybin as an agonist of 5-HT2A receptors located in cortical pyramidal cells exerted a significant effect on glutamate (GLU) extracellular levels in both the frontal cortex and hippocampus. Increased GLU release from pyramidal cells in the prefrontal cortex results in increased activity of γ-aminobutyric acid (GABA)ergic interneurons and, consequently, increased release of the GABA neurotransmitter. It seems that this mechanism appears to promote the antidepressant effects of psilocybin. By interacting with the glutamatergic pathway, psilocybin seems to participate also in the process of neuroplasticity. Therefore, the aim of this mini-review is to discuss the available literature data indicating the impact of psilocybin on glutamatergic neurotransmission and its therapeutic effects in the treatment of depression and other diseases of the nervous system.

## Introduction

Although the hallucinogenic effects of psilocybin had been known for hundreds of years, it was not until the 1960s that synthetic psilocybin produced by Sandoz was used and sold for experimental purposes, but pharmacological data were scarce [1]. The 1990s saw significantly expanded knowledge about the pharmacokinetics and pharmacodynamics of psilocybin. Pharmacological data collected over decades have certainly contributed to the revival of clinical trials of psychedelic substances, including psilocybin. In the last 20 years, more than a hundred clinical studies have been conducted to investigate the therapeutic effects of psilocybin in the treatment of brain disorders including major depressive disorder, treatment-resistant depression, anxiety symptoms, or obsessive-compulsive disorder [2]. To date, these studies have provided evidence that psilocybin, alongside ketamine, can be used as a fast-acting antidepressant [3, 4]. Therefore, psilocybin is a new and promising drug for several mental disorders that are difficult to treat due to insufficient effectiveness or resistance to currently available drugs. However, despite the use of psilocybin in clinical studies, its exact mechanism of interaction with glutamate (GLU) has not been fully understood. To date, few preclinical studies on animal models and clinical studies suggest its interaction with this neurotransmitter. Understanding the exact direction of psilocybin’s interaction with GLU is crucial from the point of view of introducing this substance in the treatment of neuropsychiatric disorders. This especially applies to diseases that are characterized by disturbances in the proper functioning of this most stimulating neurotransmitter.

Due to the large gap in the state of knowledge about the interaction of psilocybin with the glutamatergic pathway, this paper aims to summarize the current experimental data on this topic. This mini-review also considers the use of this substance as a psychedelic drug in the treatment of depression, its potential involvement in the process of neuroplasticity, along with a brief summary of its possible applications in clinical trials of other diseases of the nervous system.

## Pharmacology of psilocybin

Psilocybin (4-phosphoryloxy-N, N-dimethyltryptamine) belongs to the group of hallucinogenic tryptamines, naturally occurring of some species of mushrooms of the genus *Psilocybe* also known as “magic mushrooms” [2]. In humans, psilocybin is rapidly dephosphorylated to psilocin (4-hydroxy-N, N-dimethyltryptamine) by alkaline phosphatase in the liver and by a non-specific esterase in the intestinal mucosa [5]. In animals such as rodents, psilocybin is completely converted to psilocin before it enters the bloodstream [6]. Psilocin is the main pharmacologically psychoactive substance, which means that psilocybin acts as its prodrug. Both above-mentioned substances have similar psychotropic effects on humans at equimolar concentrations [7]. Furthermore, inhibition of alkaline phosphatase completely suppresses the psychoactive effects of psilocybin administration [8]. Moreover, psilocin has been experimentally shown to produce psychotomimetic effects and, unlike psilocybin, can cross the blood-brain barrier [2].

The effect of psilocybin depends on the mental attitude, body weight, metabolism, and tolerance level [2, 3]. The peak effect of psilocybin at doses of 2 to 6gdried *Psilocybe cubensis* mushroom (whole or powdered), which produces psychedelic effects, has been estimated at 30 to 60 min, subsiding approximately six hours after oral ingestion. In turn, doses of 3–5 mg cause sympathomimetic, but not hallucinogenic, effects [2, 9]. According to research conducted at the Johns Hopkins University School of Medicine, psilocybin administered in doses of 20–30 mg is directly associated with sustained positive effects on behavior and mood beyond 14 months [10]. Brown et al. [11] also postulated that an oral dose of 25 mg psilocybin, equivalent to approximately 0.3 mg/kg body weight, may fall within the therapeutic window. However, more clinical studies are needed to clarify the effective dose range.

Psilocybin, as one of the classic psychedelics, interacts with serotonin receptors in the brain with the highest affinity for the serotonin receptor type 2 A (5-HT2A) receptors and, to a lesser extent, for serotonin receptor type 1 A (5-HT1A) and serotonin receptor type 2 C (5-HT2C) receptors [12]. The 5-HT2A receptor belongs to the family of G protein-coupled receptors (GPCR), specifically Gq/11, the activation of which stimulates phospholipase C (PLC) and then protein kinase C (PKC), which leads to extracellular calcium release and subsequent membrane depolarization [13]. Another pathway of 5-HT2A receptor activation involves the stimulation of phospholipase A2 (PLA 2), which can hydrolyze phospholipids and produce free arachidonic acid [14].

## Hallucinogenic-like effect of psilocybin in rodents

It is now believed that activation of the 5-HT2A receptor is responsible for the psychedelic effects of psilocybin [12]. This is confirmed by the effect of ketanserin, which, as a 5-HT2A receptor antagonist, reverses the effects caused by psilocybin [15]. In animal models, hallucinogenic effects can be observed in mice as rapid side-to-side head movement (head shaking; HTR) or in rats as wet-dog shaking (WDS). Psychedelic 5-HT2A receptor agonists have been reported to cause rapid, rhythmic head movements, whereas non-psychedelic 5-HT2A receptor agonists, such as lisuride or ergotamine, do not cause head shaking [16]. Therefore, this test has a high predictive value that can be used to select a therapeutic dose, thereby eliminating the psychotomimetic effect of psychedelics.

The impact of other serotonin receptors on the psychoactive effects of psilocybin has not yet been proven.

## Hallucinogenic effects of psilocybin in humans

The use of psilocybin in humans leads to an altered state of consciousness including distortion of the subjective experience of oneself i.e. hallucinations [17]. This process leads to disruption of the sense of the boundaries of one’s world, thus increasing the sense of unity with others and the environment. Generally, hallucinations lead to positive changes in users’ consciousness, but the direction of changes leading to negative self-perception is also possible [17]. Unfortunately, the direction of changes in users’ consciousness is currently unknown which poses a significant problem in defining psilocybin as a therapeutic agent for mental illness.

Psilocybin-induced hallucinogenic effects in humans are the result of induced frontal hyperfrontality, which in turn mediates antidepressant and anxiolytic effects [3]. The fact that psilocybin causes cortical activation has been demonstrated by an increased level of cerebral glucose metabolism or an increased rate of glucose metabolism in prefrontal areas [18, 19]. Furthermore, psilocybin consumption has been shown to lead to significant 5-HT2A receptor saturation in cortical areas, which is closely related to subjective ratings of psilocybin’s psychedelic effects [17].

It has been proposed that psilocybin exerts its antidepressant effects by deactivating or normalizing the hyperexcitability of the medial prefrontal cortex (mPFC) [17, 20, 21].

Studies using light microscopic autoradiography have shown high concentrations of 5-HT2A receptors on pyramidal neurons of layer III and V cortical areas [22]. Activation of 5-HT2A receptors located on mPFC pyramidal cells leads to changes in functional connectivity between cortical and limbic areas, including the hippocampus (HP), thalamus, and structures such as the amygdala and claustrum. These structures play a fundamental role in cognitive processes and emotion processing [23, 24]. Psilocybin-induced changes in the connectivity of the mentioned structures lead to the breakdown of associative networks and lack of integration of sensory networks [17]. It is currently also postulated that the psychotomimetic effects of psilocybin are caused by its interactions with thalamocortical feedback loops.

## GLU and γ-aminobutyric acid (GABA)

GLU is the main excitatory neurotransmitter system for over 50% of neurons in the central nervous system (CNS), participating in most of the information-processing routes that occur there [25]. It plays an important role in the process of maturation and proliferation of neurons, in learning processes, and in the creation of memory traces. It is also responsible for brain plasticity and the survival of progenitor cells, as shown by the results of many studies on rodents [25, 26]. GLU in target cells binds to many specific protein complexes that include the primary types of glutamate receptors (GLUR): ionotropic glutamate receptors (iGLUR) and metabotropic glutamate receptors (mGLUR). The former are divided into three groups: Kaine, α-amino-3-hydroxy-5-methyl-4-isoxazole propionic acid receptor (AMPA), and N-methyl-D-aspartate receptor (NMDA), these are ion channels that cause depolarization of the neuronals’ cell membrane, and their role is to control the flow of cations through the plasma membrane [27]. mGLUR has been identified in all brain structures, including the limbic system. Initially, they were divided into three subfamilies based on amino acid sequence homology, the similarity of intracellular signals, and pharmacological properties: Group I mGluR (metabotropic glutamate receptors type 1 and 5; mGLU1R, mGLU5R), Group II mGLUR (metabotropic glutamate receptors type 2 and 3; mGLU2R, mGLU3R), Group III mGLUR (metabotropic glutamate receptors type 4,6,7 and 8; mGLU4R, mGLU6R, mGLU7R and mGLU8R). Their common feature is directly related to the transduction of intracellular signals activating membrane G proteins that regulate the release of CNS neurotransmitters [28, 29].

It should be emphasized that the synthesis of the inhibitory neurotransmitter, GABA, requires the enzyme glutamic acid decarboxylase (GAD) and GLU as a substrate. To date, GABA has been localized in most presynaptic and postsynaptic terminals of nerve cells. Abnormalities in its release are associated with the development of many neurological and neuropsychological disorders [30, 31]. In addition to its function as an inhibitory neurotransmitter, GABA also acts as a trophic factor regulating: proliferation, neuroblast migration, dendrite growth, and synapse formation during early embryonic development [32]. This neurotransmitter, like GLU, is involved in processes related to brain plasticity, such as the learning process, the creation of memory traces, or even the ability to move [33].

## Psilocybin: interactions between 5-HT2A and the glutamatergic pathway

Clinical studies provide evidence for the interaction of the serotonergic system with the glutamatergic system. Activation of 5-HT2A receptors leads to a GLU-dependent increase in the activity of pyramidal neurons in the prefrontal cortex, thereby modulating the activity of the prefrontal network, as confirmed by clinical studies and animal models [17, 34]. Our research confirms this relationship. We have shown that psilocybin at doses of 2 mg/kg and 10 mg/kg increases the extracellular release of 5-HT in the prefrontal cortex, which is most likely related to the strong activation of 5-HT2A receptors located on the cortical projections to the dorsal raphe nuclei (DRN) [34]. A similar profile of changes in the frontal cortex (FC) was reported by Sakashita et al. [35] after the administration of 10 mg/kg psilocin.

The effect of psychedelics on glutamatergic activity is reversed by agonists of inhibitory metabotropic group II/III GLUR located presynaptically as well as antagonists of ionotropic AMPA/kainate receptors acting postsynaptically. This is a direct confirmation of the interaction of the serotonergic and glutamatergic pathways, which may be the route of action of psychedelics and a potential mechanism underlying the therapeutic effects of these compounds [36].

Martin and Nichols [37] found that in rats, structures such as the mPFC and claustrum contained neurons with a high density of 5-HT2A receptors, which means that these cells are highly sensitive to the effects of psychedelics, including psilocybin, which shows a strong agonism towards this group of receptors. In particular, pyramidal cells of layer V of the mPFC seem to be the main source of 5-HT2A receptors and therefore the main target of psilocybin action [38, 39].

The mentioned separate group of neurons most likely acts as a “specific activator” of excitatory transmission, which leads to increased activity of subcortical areas. However, this method of activation is not characterized by the constant generation of action potentials. The release of GLU from this small group of thalamocortical afferents results in their recurrent activity, which leads to changes in the activity of other types of neurons, including GABAergic interneurons [36, 40]. 5-HT2A receptors are colocalized with 5-HT1A receptors on GABAergic interneurons, for which psilocybin has lower affinity [41]. This means that the subsequent cellular response is determined by the summation of the effects of inhibitory 5-HT1A and excitatory 5-HT2A receptors. In this way, psilocybin can indirectly modulate the activation of limbic system structures such as the HP, nucleus accumbens, and the amygdala [24].

In our studies, we demonstrated increased release of extracellular GLU and GABA in the FC of rats after administration of psilocybin at a dose of 10 mg/kg. The low dose of 2 mg/kg had a very weak effect on the release of both these neurotransmitters [34]. Similar observations were reported by Mason et al., [17], namely psilocybin caused an increase in GLU and GABA levels in the human mPFC. These results therefore suggest that psilocybin participates in cortical activity by increasing the release of GLU from pyramidal cells as well as stimulating GABAergic interneurons to increase the release of the neurotransmitter GABA. The changes described above are most likely related to the expression of 5-HT2A receptors on both glutamatergic and GABAergic neurons in the cortex [34].

Furthermore, psilocybin’s effects on the glutamatergic system in the FC appear to produce a distortion in the subjective experience of self, the so-called “ego dissolution.” As suggested by Mason et al., [17], increased GLU release in the mPFC is responsible for the negative experience of ego dissolution. The same authors also suggested that reduced GLU release in the HP caused a positive experience of ego dissolution. Comparing this to our results, the dose of 2 mg/kg incited an advantage of inhibitory transmission over excitatory glutamatergic transmission in the rat HP [24]. This is most likely related to the agonism of 5-HT2A receptors located on GABAergic interneurons, which leads to the reduced activation of pyramidal neurons in this area. In our studies, a balance between inhibitory transmission and excitatory transmission was also demonstrated in the HP of rats under the influence of 10 mg/kg psilocybin [24]. This dose, therefore, appears to promote the interaction of 5-HT2A receptors with 5-HT1A receptors, which have been experimentally determined to be highly concentrated in the HP [42, 43].

Accurate determination of the GLU/GABA ratio in both the mPFC and HP seems to be an essential predictor for determining the dose of psilocybin used in the treatment of mental disorders. Research is currently underway to eliminate the side effects of psilocybin, most often referred to as the so-called “bad trip” [10, 44]. Taking into account the above reports, future research should focus on the precise determination of the interaction of 5-HT2A and 5-HT1A receptors to be able to possibly predict its therapeutic direction and thus eliminate its side effects.

## Psilocybin and neuroplasticity

The increase in glutamatergic signaling under the influence of psilocybin is reflected in its potential involvement in the neuroplasticity process [45, 46]. An increase in extracellular GLU increases the expression of brain-derived neurotrophic factor (BDNF), a protein involved in neuronal survival and growth. However, too high amounts of the released GLU can cause excitotoxicity, leading to the atrophy of these cells [47]. The increased BDNF expression and GLU release by psilocybin most likely leads to the activation of postsynaptic AMPA receptors in the prefrontal cortex and, consequently, to increased neuroplasticity [2, 48]. However, in our study, no changes were observed in the synaptic iGLUR AMPA type subunits 1 and 2 (GluA1 and GluA2)after psilocybin at either 2 mg/kg or 10 mg/kg.

Other groups of GLUR, including NMDA receptors, may also participate in the neuroplasticity process. Under the influence of psilocybin, the expression patterns of the c-Fos (cellular oncogene c-Fos), belonging to early cellular response genes, also change [49]. Increased expression of c-Fos in the FC under the influence of psilocybin with simultaneously elevated expression of NMDA receptors suggests their potential involvement in early neuroplasticity processes [37, 49]. Our experiments seem to confirm this. We recorded a significant increase in the expression of the GluN2A 24 h after administration of 10 mg/kg psilocybin [34], which may mean that this subgroup of NMDA receptors, together with c-Fos, participates in the early stage of neuroplasticity.

As reported by Shao et al. [45], psilocybin at a dose of 1 mg/kg induces the growth of dendritic spines in the FC of mice, which is most likely related to the increased expression of genes controlling cell morphogenesis, neuronal projections, and synaptic structure, such as early growth response protein 1 and 2 (Egr1; Egr2) and nuclear factor of kappa light polypeptide gene enhancer in B-cells inhibitor alpha (IκBα). Our study did not determine the expression of the above genes, however, the increase in the expression of the GluN2A subunit may be related to the simultaneously observed increase in dendritic spine density induced by activation of the 5-HT2A receptor under the influence of psilocybin [34].

The effect of psilocybin in this case can be compared to the effect of ketamine an NMDA receptor antagonist, which is currently considered a fast-acting antidepressant, which is related to its ability to modulate glutamatergic system dysfunction [50, 51]. The action of ketamine in the frontal cortex depends on the interaction of the glutamatergic and GABAergic pathways. Several studies, including ours, seem to confirm this assumption. Ketamine shows varying selectivity to individual NMDA receptor subunits [52]. As a consequence, GLU release is not completely inhibited, as exemplified by the results of Pham et al., [53] and Wojtas et al., [34]. Although the antidepressant effect of ketamine is mediated by GluN2B located on GABAergic interneurons, but not by GluN2A on glutamatergic neurons, it cannot be ruled out that psilocybin has an antidepressant effect using a different mechanism of action using a different subgroup of NMDA receptors, namely GluN2A.

All the more so because the time course of the process of structural remodeling of cortical neurons after psilocybin seems to be consistent with the results obtained after the administration of ketamine [45, 54]. Furthermore, changes in dendritic spines after psilocybin are persistent for at least a month [45], unlike ketamine, which produces a transient antidepressant effect. Therefore, psychedelics such as psilocybin show high potential for use as fast-acting antidepressants with longer-lasting effects. Since the exact mechanism of neuroplasticity involving psychedelics has not been established so far, it is necessary to conduct further research on how drugs with different molecular mechanisms lead to a similar end effect on neuroplasticity. Perhaps classically used drugs that directly modulate the glutamatergic system can be replaced in some cases with indirect modulators of the glutamatergic system, including agonists of the serotonergic system such as psilocybin. Ketamine also has several side effects, including drug addiction, which means that other substances are currently being sought that can equally effectively treat neuropsychiatric diseases while minimizing side effects.

As we have shown, psilocybin can enhance cognitive processes through the increased release of acetylcholine (ACh) in the HP of rats [24]. As demonstrated by other authors [55], ACh contributes to synaptic plasticity. Based on our studies, the changes in ACh release are most likely related to increased serotonin release due to the strong agonist effect of psilocybin on the 5-HT2A receptor [24]. 5-HT1A receptors also participate in ACh release in the HP [56]. Therefore, a precise determination of the interaction between both types of receptors in the context of the cholinergic system will certainly contribute to expanding our knowledge about the process of plasticity involving psychedelics.

## Conclusions and future perspectives

Psilocybin, as a psychedelic drug, seems to have high therapeutic potential in neuropsychiatric diseases. The changes psilocybin exerts on glutamatergic signaling have not been precisely determined, yet, based on available reports, it can be assumed that, depending on the brain region, psilocybin may modulate glutamatergic neurotransmission. Moreover, psilocybin indirectly modulates the dopaminergic pathway, which may be related to its addictive potential. Clinical trials conducted to date suggested the therapeutic effect of psilocybin on depression, in particular, as an alternative therapy in cases when other available drugs do not show sufficient efficacy. A few experimental studies have reported that it may affect neuroplasticity processes so it is likely that psilocybin’s greatest potential lies in its ability to induce structural changes in cortical areas that are also accompanied by changes in neurotransmission.

Despite the promising results that scientists have managed to obtain from studying this compound, there is undoubtedly much controversy surrounding research using psilocybin and other psychedelic substances. The main problem is the continuing historical stigmatization of these compounds, including the assumption that they have no beneficial medical use. The number of clinical trials conducted does not reflect its high potential, which is especially evident in the treatment of depression. According to the available data, psilocybin therapy requires the use of a small, single dose. This makes it a worthy alternative to currently available drugs for this condition. The FDA has recognized psilocybin as a “Breakthrough Therapies” for treatment-resistant depression and post-traumatic stress disorder, respectively, which suggests that the stigmatization of psychedelics seems to be slowly dying out. In addition, pilot studies using psilocybin in the treatment of alcohol use disorder (AUD) are ongoing. Initially, it has been shown to be highly effective in blocking the process of reconsolidation of alcohol-related memory in combined therapy. The results of previous studies on the interaction of psilocybin with the glutamatergic pathway and related neuroplasticity presented in this paper may also suggest that this compound could be analyzed for use in therapies for diseases such as Alzheimer’s or schizophrenia. Translating clinical trials into approved therapeutics could be a milestone in changing public attitudes towards these types of substances, while at the same time consolidating legal regulations leading to their use.

## Data Availability

No datasets were generated or analysed during the current study.

## Abbreviations

5: HT1A-Serotonin receptor type 1 A
5: HT2A-Serotonin receptor type 2 A
5: HT2C-Serotonin receptor type 2 C
ACh: Acetylcholine
AMPA: α-Amino-3-hydroxy-5-methyl-4-isoxazole propionic acid receptor
BDNF: Brain-derived neurotrophic factor
c: Fos-Cellular oncogene c-Fos
CNS: Central nervous system
DRN: Dorsal raphe nucleus
Egr1: Early growth response protein 1
Egr2: Early growth response protein 2
FC: Frontal cortex
GABA: γ-Aminobutyric acid
GAD: Glutamate decarboxylase
GLU: Glutamate
GLUR: Glutamate receptor
GluA1: Glutamate receptor ionotropic AMPA type subunit 1
GluA2: Glutamate receptor ionotropic AMPA type subunit 2
GluN2A: Glutamate receptor ionotropic NMDA type subunit 2 A
GluN2B: Glutamate receptor ionotropic NMDA type subunit 2B
GPCR: G protein-coupled receptors
HP: Hippocampus
HTR: Head-twitch response
iGLUR: Ionotropic glutamate receptors
IκBα: Nuclear factor of kappa light polypeptide gene enhancer in B-cells inhibitor alpha
mGLU1: Metabotropic glutamate receptors type 1
mGLU2: Metabotropic glutamate receptors type 2
mGLU3: Metabotropic glutamate receptors type 3
mGLU4: Metabotropic glutamate receptors type 4
mGLU5: Metabotropic glutamate receptors type 5
mGLU6: Metabotropic glutamate receptors type 6
mGLU7: Metabotropic glutamate receptors type 7
mGLU8: Metabotropic glutamate receptors type 8
mGLUR: Metabotropic glutamate receptors
mPFC: Medial prefrontal cortex
NMDA: N-Methyl-D-aspartate receptor
OF: Open field test
PKC: Protein kinase C
PLA 2: Enzyme phospholipase A type 2
PLC: Enzyme phospholipase C
WDS: Wet dog shake behavior
